# Experimental sepsis in pigs—effects of vasopressin on renal, hepatic, and intestinal dysfunction

**DOI:** 10.3109/03009734.2011.650796

**Published:** 2012-08

**Authors:** Mu-Huo Ji, Jian-Jun Yang, Jing Wu, Ren-Qi Li, Guo-Min Li, Yun-Xia Fan, Wei-Yan Li

**Affiliations:** ^1^Department of Anesthesiology, Jinling Hospital, School of Medicine, Nanjing University, Nanjing, P. R. China, and; ^2^Department of Anesthesiology, Jintan Hospital, Jiangsu University, Changzhou, P. R. China

**Keywords:** Blood flow, intestine, kidney, liver, sepsis, vasopressin

## Abstract

**Introduction:**

Low-dose arginine vasopressin (AVP) has been proposed as an adjunctive vasopressor for the treatment of advanced vasodilatory shock. However, its effects on renal, hepatic, and intestinal dysfunction during sepsis remain controversial.

**Methods:**

Fecal peritonitis was induced in 20 anesthetized, invasively monitored, mechanically ventilated female pigs. Following the time point of septic shock (defined as mean artery pressure (MAP) ≤65 mmHg), animals were randomly assigned to the following groups (*n* = 10): 1) a norepinephrine group with MAP between 65 and 75 mmHg; and 2) an AVP group with a constant infusion rate of 0.5 mU.kg^-1^.min^-1^.

**Results:**

MAP, pulmonary capillary wedge pressure, hematocrit, TNF-α, IL-6, and IL-10 were similar in the two groups during the 28-h observation period. Infusion of AVP was associated with lower total norepinephrine and fluid requirements. There was a statistically significant improvement in renal function as assessed by increased urine output and renal blood flow, and decreased serum creatinine, in the AVP group when compared with the norepinephrine group (P < 0.05). Histological analyses of the intestine, liver, and kidney showed similar light microscopical appearance of the two groups. Apoptotic cells in the liver were significantly fewer in the AVP group when compared with the norepinephrine group (P < 0.05).

**Conclusion:**

An adjunctive AVP to norepinephrine infusion exhibits a favorable impact on renal function without deleterious effects on the liver and intestine in a porcine model of experimental sepsis when compared with norepinephrine infusion alone.

## Introduction

Despite a substantial improvement in diagnosis and treatment, severe sepsis and septic shock still remain among the leading causes of death in the intensive care unit (1). Hypoperfusion in organs from the splanchnic territory plays a pivotal role in the pathogenesis of multiple organ failure (2). Thus, in addition to the treatment of underlying disease and volume resuscitation, effective cardiovascular support with vasopressors is of crucial importance in the management of septic shock patients (2). Unfortunately, no clear consensus has been currently reached regarding the optimal agent to augment blood pressure in septic shock. A pressor catecholamine such as norepinephrine continues to be the standard of care in this condition. However, high doses of norepinephrine may exert some adverse effects (3), thereby perpetuating the development of multiple organ failure.

The recent Vasopressin and Septic Shock Trial (VASST) has suggested that a supplementary arginine vasopressin (AVP) infusion to norepinephrine may benefit patients with less severe septic shock (3). Indeed, infusion of low doses to compensate for a relative AVP deficiency has shown some favorable effects on hemodynamics in general in patients with advanced vasodilatory shock (4–6). However, conflicting results have been reported regarding the effects of AVP on hepatosplanchnic perfusion (2,5,7). Furthermore, its effects on inflammatory responses during sepsis remain less well studied.

Hence, the aim of the present study was to assess the effects of AVP (supplemented with norepinephrine) on hemodynamics, inflammatory responses, organ blood flow, and organ function in a porcine model of experimental sepsis when compared with norepinephrine infusion alone.

## Methods

### Animal care

The study was approved by the ethics committee of Nanjing University Medical School and was performed in accordance with the Guideline for the Care and Use of Laboratory Animals from the National Institutes of Health. Twenty female domestic pigs (weight 26–35 kg) were fasted overnight but were allowed free access to water.

### Anesthesia and surgical procedures

After induction of anesthesia with i.m. ketamine hydrochloride (20 mg kg^-1^; HenRui Co., Jiangsu, China), the pigs were placed in a supine position and the cephalic vein was cannulated with a peripheral venous catheter (18 F; PuYi Co., Shanghai, China). The animals were then orally intubated and mechanically ventilated in a controlled volume mode (Servo ventilator 900 C; Siemens-Elema, Solna, Sweden) with a positive end-expiratory pressure of 5 cmH_2_O, a tidal volume of 6–10 mL kg^-1^ min^-1^, an inspired oxygen fraction of 0.21, and an inspired time/expiratory time of 1:2. Anesthesia was maintained with continuous intravenous infusions of fentanyl (10 µg kg^-1^ h^-1^; Renfu Co., Hubei, China), propofol (2 mg kg^-1^ h^-1^; AstraZeneca, Wuxi, China), and vecuronium (0.3 mg kg^-1^ h^-1^; Renfu Co., Hubei, China). Tidal volume and respiratory rate were adjusted to maintain end-tidal carbon dioxide tension between 35 and 45 mmHg. The right femoral artery was catheterized for monitoring of arterial blood pressure and withdrawal of arterial blood samples. Through the right jugular vein, an introducer was inserted, and a 7.0 F Swan–Ganz catheter (Edwards Life Sciences, Irvine, CA, USA) was floated into the pulmonary artery under pressure waveform monitoring. A Foley catheter (14 F; PuYi Co., Shanghai, China) was inserted to collect and monitor the urine output. Body temperature was monitored by a rectally inserted probe.

Through a midline laparotomy, the hepatic, right renal, and mesenteric arteries were prepared and visualized, and hepatic, renal, and mesenteric artery blood flows were measured using ultrasonic flow probes (Transonic Systems, Ithaca, NY, USA). The cecal and ileocecal areas were identified, and, after making a 1-cm perforation in the cecal tip, spillage of fecal material (1 g.kg^-1^ of body weight) was collected in a 100 mL syringe. The cecum and abdominal cavity were then closed by fascial and cutaneous sutures. After 1 hour of stabilization and baseline data collection, peritonitis was induced by inoculating autologous feces (1 g kg^-1^) into the abdominal cavity as previously described (8).

### Experimental protocol

After the onset of septic shock (defined as mean artery pressure ≤65 mmHg), animals were randomly assigned to the following two groups (each, *n* = 10): A norepinephrine group was titrated to maintain a mean artery pressure (MAP) between 65 and 75 mmHg. An AVP group was titrated at a constant infusion rate of 0.5 mU.kg^-1^ketamine^-1^. If necessary, norepinephrine was given to maintain a MAP between 65 and 75 mmHg. Lactated Ringer's solution (10 mL kg^-1^ h^-1^; WanTong, Co., Jilin, China) and hydroxyethyl starch (5 mL kg^-1^ h^-1^; 6% hydroxyethyl starch 130/0.6; Fresennius, Beijing, China) were infused as maintenance fluid. Additional fluids (crystalloid/colloid ratio 2:1) were infused if there was an increase of the hematocrit value.

### Hemodynamic and organ blood flow measurements

Cardiac output (CO), heart rate (HR), pulmonary capillary wedge pressure (PCWP), MAP, hepatic, renal, and mesenteric artery blood flows, and arterial lactate concentrations were recorded at the following time points: baseline, shock time, 4, 8, and 12 h after septic shock.

### Histological analyses and apoptosis assessment

Animals surviving 12 h after the onset of septic shock were killed under deep anesthesia with a lethal dose of 10% potassium chloride. Tissue samples of intestine, liver, and kidney were immediately taken and stored in formalin. A pathologist unaware of the study protocol analyzed the tissue samples using light microscopy. The amount of apoptotic nuclei was determined in liver tissue sections using a TUNEL assay (Boehringer, Mannheim, Germany). Fluorescein-conjugated dUTP incorporated in nucleotide polymers was detected and quantified using fluorescence microscopy (Zeiss LSM 410, Wetzlar, Germany). Only nuclear staining was considered positive.

### Other laboratory analyses

Serum concentrations of alanine aminotransferase (ALT), aspartate aminotransferase (AST), bilirubin, and creatinine were determined by using assay kits (Xie He Co., Beijing, China). Intestinal tissue concentrations of inflammatory cytokines (TNF-α, IL-6, IL-10) were quantified using specific ELISA kits for swine (4A Biotech Co., Beijing, China). Arterial total nitrate/nitrite concentrations (NO_X_a, a surrogate of NO) were measured using a Griess reagent (Cayman Chemical Nitrite/Nitrite Assay Kit; Cayman Chemical Co., Ann Arbor, MI, USA). Serum concentrations of high mobility group box 1 (HMGB1) were assayed by a sandwich method (Shino-Test Corporation, Tokyo, Japan).

### Statistical analysis

Kolmogorov–Smirnov test was applied to determine if the collected data formed a normal distribution, and normality was obtained for all main measurements. Data collected from experiments forming normal distributions were expressed as mean ± standard deviation (SD). Missing values were accounted for using the last-observation-carried-forward method. Intergroup comparisons of the parameters for multiple time points were performed using analysis of variance (ANOVA) for repeated measurements. Student's unpaired *t* test was used to compare parameters for single time points between the two groups. Statistical analysis was performed using the SPSS 16.0 software for Windows (SPSS, Chicago, IL, USA). *P* < 0.05 was considered to be statistically significant.

## Results

Four animals in the norepinephrine group and three in the AVP group died before the end of the study due to refractory arterial hypotension. There were no statistically significant differences in any of the variables measured at baseline or when septic shock (ST) was diagnosed. MAP, PCWP, and hematocrit were similar in the two groups (*P* > 0.05). Likewise, mean body weight and time to onset of septic shock did not differ (data not shown).

### Hemodynamics and metabolic changes

Animals receiving AVP required significantly less norepinephrine support (1.1 ± 0.2 mg kg^-1^) when compared with the norepinephrine animals (1.6 ± 0.3 mg kg^-1^) (*P* < 0.05). Total fluid requirement was lower in the AVP group (20.8 ± 1.1 mL kg^-1^ h^-1^) when compared with the norepinephrine group (23.6 ± 1.2 mL kg^-1^ h^-1^) (*P* < 0.05). HR and CO in the AVP group were lower than in the norepinephrine group 4 h and 8 h after ST (*P* < 0.05) (Table I). There was, however, no difference in stroke volume. Renal artery blood flow increased in the AVP group 8 h and 12 h after onset of the septic shock when compared with the norepinephrine group (*P* < 0.05). Hepatic and mesenteric blood flows were comparable as well (Table I).

**Table I.** Changes in hemodynamics and renal, hepatic, and mesenteric arterial blood flow.

Variable	Time/Group	BL	ST	4 h	8 h	12 h
MAP (mmHg)	NE	97 ± 11	59 ± 1	71 ± 3	70 ± 3	69 ± 3
	AVP	102 ± 13	60 ± 1	69 ± 3	69 ± 3	71 ± 3
HR (beats min^-1^)	NE	87 ± 10	103 ± 13	132 ± 11	143 ± 17	145 ± 21
	AVP	92 ± 11	105 ± 13	111 ± 10^a^	122 ± 13^a^	137 ± 16
CO (mL kg^-1^ min^-1^)	NE	99 ± 8	108 ± 9	159 ± 21	147 ± 16	139 ± 13
	AVP	101 ± 10	105 ± 7	117 ± 11^a^	124 ± 13^a^	131 ± 10
PCWP (mmHg)	NE	9 ± 2	13 ± 3	14 ± 3^b^	15 ± 3	16 ± 2
	AVP	10 ± 3	13 ± 3	15 ± 3^b^	16 ± 2	17 ± 2
SV (mL kg^-1^)	NE	1.1 ± 0.1	1.0 ± 0.1	1.2 ± 0.2	1.0 ± 0.1	1.0 ± 0.1
	AVP	1.1 ± 0.1	1.0 ± 0.1	1.1 ± 0.1	1.0 ± 0.1	1.0 ± 0.1
RBF (% of BL)	NE	100 ± 0	93 ± 6	104 ± 9	93 ± 8	84 ± 10
	AVP	100 ± 0	87 ± 8	99 ± 7	111 ± 9^a^	116 ± 11^a^
HBF (% of BL)	NE	100 ± 0	103 ± 8	116 ± 14	109 ± 17	117 ± 11
	AVP	100 ± 0	106 ± 6	107 ± 11	117 ± 21	109 ± 13
MBF (% of BL)	NE	100 ± 0	93 ± 6	119 ± 15	109 ± 13	81 ± 11
	AVP	100 ± 0	97 ± 8	108 ± 11	98 ± 11	88 ± 13
Hematocrit (%)	NE	29 ± 4	30 ± 3	31 ± 3	32 ± 3	33 ± 3
	AVP	28 ± 3	29 ± 3	30 ± 3	31 ± 4	32 ± 4

^a^ *P* < 0.05 versus NE group. AVP = arginine vasopressin; BL = baseline; CO = cardiac output; HBF = hepatic blood flow; HR = heart rate; MBF = mesenteric blood flow; NE = norepinephrine; PCWP = pulmonary capillary wedge pressure; RBF = renal blood flow; ST = shock time; SV = Stroke volume.

### Renal and hepatic function

Sepsis-induced kidney dysfunction was indicated by increased serum creatinine concentrations and decreased urine output (Table II). When compared with the norepinephrine group, AVP-treated animals had a much improved renal function as assessed by increased urine output and decreased serum creatinine (*P* < 0.05). Although serum bilirubin, ALT, and AST concentrations were significantly increased in both groups, there was no statistically significant difference in these parameters between the two groups (*P* > 0.05). Likewise, the increase in serum lactate concentration was also less pronounced in the AVP group (*P* < 0.05) (Table II).

**Table II.** Serum parameters of liver and kidney functions and lactate concentrations.

Variable	Time/Group	BL	ST	6 h	12 h
AST (U L^-1^)	NE	71 ± 45	91 ± 27	102 ± 34	163 ± 59
	AVP	79 ± 25	103 ± 44	104 ± 43	167 ± 44
ALT (U L^-1^)	NE	18 ± 10	22 ± 9	26 ± 8	29 ± 10
	AVP	26 ± 8	24 ± 9	27 ± 8	23 ± 8
Bilirubin (mg dL^-1^)	NE	0.7 ± 0.2	0.8 ± 0.2	1.0 ± 0.3	1.9 ± 0.6
	AVP	0.6 ± 0.4	0.9 ± 0.3	1.1 ± 0.5	2.1 ± 0.5
Creatinine (mg dL^-1^)	NE	55 ± 11	82 ± 14	98 ± 13	208 ± 44
	AVP	52 ± 9	67 ± 7	98 ± 9	165 ± 32^a^
AUO (mL)	NE	124 ± 79	1733 ± 168	2030 ± 122	2076 ± 103
	AVP	125 ± 77	1729 ± 210	2124 ± 217	2467 ± 167^a^
Lactate concentration (mmol L^-1^)	NE	0.9 ± 0.3	1.9 ± 0.3	2.5 ± 0.5	3.7 ± 0.5
	AVP	1.0 ± 0.4	2.1 ± 0.4	2.3 ± 0.4	2.8 ± 0.4^a^

^a^ *P* < 0.05 versus NE group. ALT = alanine aminotransferase; AST = aspartate aminotransferase; AUO = accumulated urine output; AVP = arginine vasopressin; BL = baseline; NE = norepinephrine; ST = shock time.

### Inflammatory mediators

Systemic inflammation was evidenced by increases over time in serum TNF-α, IL-6, IL-10, and HMGB1 in both groups (Table III). However, serum concentrations of TNF-α, IL-6, IL-10, and HMGB1 were comparable in the two groups (Table III). Arterial total nitrate/nitrite concentrations were lower in the AVP group when compared with the norepinephrine group 4 and 12 h after the septic shock (*P* < 0.05) (Table III). The intestinal IL-6 concentration was significantly lower in the AVP group (1.2 ± 0.5 pg mg^-1^) when compared with the norepinephrine group (2.0 ± 0.8 pg mg^-1^) (*P* < 0.05). However, there were no differences with regard to intestinal TNF-α and IL-10 concentrations (data not shown).

**Table III.** Changes in serum TNF-α, IL-6, IL-10, NO_X_a, and HMGB1.

Variable	Time/Group	BL	ST	4 h	12 h
TNF-α (pg mL^-1^)	NE	11 ± 4	3546 ± 872	853 ± 217	68 ± 14
	AVP	16 ± 6	3161 ± 919	448 ± 113	63 ± 1
IL-6 (ng mL^-1^)	NE	ND	13.3 ± 3.8	9.8 ± 2.9	4.2 ± 1.3
	AVP	ND	15.6 ± 4.3	10.7 ± 3.5	3.7 ± 1.4
IL-10 (pg mL^-1^)	NE	6 ± 2	134 ± 23	94 ± 24	43 ± 14
	AVP	5 ± 2	152 ± 44	89± 18	54 ± 11
NO_X_a (mol L^-1^)	NE	16 ± 5	23 ± 8	46 ± 7	49 ± 8
	AVP	19 ± 5	22 ± 6	30 ± 6^a^	33 ± 6^a^
HMGB1 (μg L^-1^)	NE	ND	5 ± 1	17 ± 4	28 ± 5
	AVP	ND	6 ± 2	15 ± 3	19 ± 4^a^

^a^ *P* < 0.05 versus NE group. AVP = arginine vasopressin; BL = baseline; ND = not detected; NE = norepinephrine; ST = shock time.

### Histological analyses

**Figure 1.**
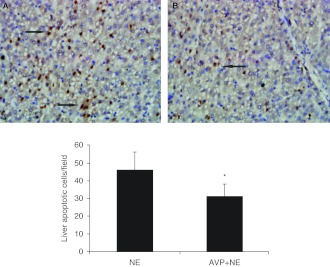
Determination of apoptosis by the TUNEL method. Numbers of TUNEL-stained cells counted in five random microscopic fields in both groups. Results were expressed as mean of TUNEL-stained cells found per field. (NE = norepinephrine; AVP = arginine vasopressin).

Histological analyses of the intestine, liver, and kidney showed moderate to severe neutrophil accumulation and edema formation. There were, however, no differences between the two groups (data not shown). However, the AVP group had less TUNEL-positive cells in the liver when compared with the norepinephrine group (Figure 1).

## Discussion

The major finding of this study was that AVP infusions in pigs with experimental sepsis at a dose of 0.5 mU.kg^-1^kg^-1^min^-1^ supplemented with norepinephrine improved renal function without deleterious effects on the liver and intestine when compared with norepinephrine infusion alone. It has been demonstrated that AVP restores the vascular tone by modulation of potassium–adenosine triphosphate channels, modulation of nitric oxide, and potentiation of adrenergic and other vasoconstrictor agents via activation of vasopressin (V_1_) receptors in catecholamine-resistant septic shock patients (3,6). On the other hand, increased doses of norepinephrine infusion may sometimes be ineffective in maintaining arterial pressure in septic shock. Splanchnic circulation is jeopardized during septic shock (9); however, covert tissue dysoxia may persist even after fluid optimization and correction of hypotension with vasoactive agents (2,10). A previous study has demonstrated that low-dose AVP supplemented with norepinephrine is safe with respect to visceral organ function and tissue integrity in a model of fecal peritonitis-induced septic shock (11). Consistent with this, we observed that AVP infusion increased renal arterial blood flow, while hepatic and mesenteric blood flow remained unchanged when compared with norepinephrine infusion alone. Our results were in contrast to a study by Martikainen et al. (2), in which they showed that using vasopressin as a monotherapy in septic shock is associated with blood flow redistribution and heterogeneous metabolic changes within splanchnic tissues. However, it is worth noting that AVP was used alone rather than as a supplementary vasopressor. It is therefore conceivable that increasing amounts of AVP appear to provide the intended effect on MAP or an organ's ‘perfusion pressure’. It is possible that the higher doses may induce more adverse effects and actually reduce an organ's ‘perfusion flow’, therefore counterbalancing the beneficial effects of low-dose AVP (5). These results suggest that low-dose vasopressin replacement therapy would be reasonable during septic shock.

During sepsis, there is often an enhanced production of pro-inflammatory mediators that can cause multiple organ failure. The intestine plays an important role in priming neutrophils and the release of inflammatory mediators during sepsis, followed by the deranged function of the intestine mucosa barrier and subsequent increased permeability (12), which in turn can lead to multiple organ failure (13). A previous study has suggested that IL-6 is involved in the loss of mucosal integrity (14). The lower intestinal concentration of IL-6 observed in the AVP group suggested protective effects of the combination therapy. The result was supported by other studies on IL-6 production (15), in which it was found that AVP reduced the IL-6 concentration in response to the endotoxin stimulus. Moreover, it has been shown that increased inducible nitric oxide synthase-dependent nitride oxide production decreases the expression of tight junction proteins and tight junction localization in endotoxemia mice (16). More recently, Westphal et al. (17) even showed that AVP infusion at a dose of 0.02 IU min^-1^ reduced nitrosative stress and improved cardiopulmonary functions in sheep with acute lung injury. In addition, it has recently been demonstrated that HMGB1 is an important late-phase mediator in the pathogenesis of sepsis. Thus, it was suggested that the serum peak HMGB1 concentrations were associated with the duration of the systemic inflammatory response syndrome and postoperative pulmonary dysfunction (18). Consistent with these observations, we noticed that the serum NO_X_a and HMGB1 concentrations were lower after AVP infusion, which may provide additional evidence for the value of AVP in the treatment of septic shock. Although there was no difference in intestinal concentrations of TNF-α or IL-10 and intestinal morphology as well between the two groups, we cannot exclude that the effects may be masked by the local, severe insult. An endotoxin model might have produced different results.

Notably, renal function, as assessed by urine output and serum creatinine, was significantly improved in the AVP group as compared with the norepinephrine group. Indeed, the diuretic effect of AVP has been confirmed in previous studies (19,20). One possible explanation is that AVP exhibits selective vasoconstriction properties on the efferent arteriole, while norepinephrine increases the resistance in both afferent and efferent glomerular arterioles. Recently, Guzman et al. (21) showed that AVP administration effectively restores renal blood flow with comparable systemic and splanchnic effects when compared to norepinephrine alone in a rat model of endotoxin shock. Hence, for similar MAP, replacement of norepinephrine with AVP may maintain the glomerular filtration rate. On the other hand, it should be noted that the glomerular filtration rate is disproportionally reduced as compared to renal blood flow, highlighting the fact that hemodynamic factors are only some of the mechanisms responsible for renal function alteration during sepsis (22). Furthermore, systemic and local inflammatory mediators can also provoke renal dysfunction even in the absence of any obvious hemodynamic disturbance (23). Therefore, we may speculate that the beneficial effects of AVP on renal function may be related to improved perfusion, while possible anti-inflammatory activities may also be involved. Furthermore, the attenuated serum lactate concentrations observed in the AVP group suggested an enhanced tissue perfusion. Thus, the decrease in lactate concentrations could be, at least in part, attributed to the improvement in renal function, although most lactate is cleared by the liver.

Previous studies have demonstrated that AVP infusion is associated with a deterioration of liver function, which may be related to an AVP-induced reduction of hepatic blood flow (24,25). However, we observed that AVP infusion was not associated with a decreased hepatic arterial blood flow. In addition, AST, ALT, and bilirubin were comparable, which suggested that the hepatosplanchnic perfusion was not deteriorated in the AVP group. Furthermore, the finding of fewer TUNEL-positive cells in the livers of the AVP-treated animals suggested a protective effect of the combination therapy.

There are some obvious limitations in the present study. First, neither causal therapy nor antibiotics were used, which obviously does not reflect the clinical situation. Second, due to the use of multiple statistical testing of numerous variables measured, the risk of false positive results should be taken into consideration. Finally, the limited experimental duration precludes any conclusion as to whether combination therapy is beneficial in a longer time perspective or even may lead to a subsequent deterioration.

In conclusion, our study provides evidence that an adjunctive AVP infusion to norepinephrine in a porcine model of experimental sepsis improves renal function without deleterious effects on intestine and liver functions when compared with norepinephrine administration alone. However, more studies are needed to define the efficacy and long-term benefits of the combination therapy.
